# Management of Idiopathic Granulomatous Mastitis: Effectiveness of a Steroid-Free Regimen Using *Tinospora cordifolia*—A Single-Institution Experience

**DOI:** 10.1155/tbj/2997891

**Published:** 2025-01-22

**Authors:** Ankita Das Sheth, Shalaka Joshi, Arul Kumar, Nita Nair, Tanuja Shet, Ayushi Sahay, Palak Thakkar, Purvi Haria, Aparna Katdare, Vani Parmar, Sangeeta Desai, Rajendra Badwe

**Affiliations:** ^1^Department of Surgical Oncology, Breast Services, Tata Memorial Centre and Homi Bhabha National Institute, Mumbai, India; ^2^Department of Pathology, Breast Services, Tata Memorial Centre and Homi Bhabha National Institute, Mumbai, India; ^3^Department of Radiodiagnosis, Breast Services, Tata Memorial Centre and Homi Bhabha National Institute, Mumbai, India

**Keywords:** idiopathic granulomatous mastitis, immunomodulators, Tinosporin

## Abstract

**Introduction:** Idiopathic granulomatous mastitis (IGM) is a benign, chronic inflammatory disease with no effective treatment and high relapse rate. The pathophysiology is poorly understood. Tinosporin, an immunomodulator obtained from *Tinospora cordifolia*, is known to be useful in treating immune-mediated diseases. We report our experience of using Tinosporin for IGM and the effectiveness of this “steroid-free” regimen.

**Methods:** We analysed the clinicopathological characteristics of patients diagnosed with IGM on histopathology during January 2018 and December 2022. Tinosporin tablet (500 mg Guduchi stem extract) was prescribed for 3–6 months; data were collected from electronic medical records and analysed in SPSS v-29.

**Results:** Of 315 patients, 132 had complete clinical records. Median age was 39 years (25–77), and 107 (81.7%) were premenopausal. Seventy-eight (59.09%) had clinical suspicion of malignancy. On imaging, 84 (63.64%) were BIRADS 4/5 lesions. Empirical broad-spectrum antibiotics were prescribed to 101 patients. Tinosporin tablets were prescribed to 91 patients. Symptomatic response was seen in 72 (79.12%). Five patients did not achieve response, while 14 patients (15.38%) were lost to follow-up. At a median follow-up of 36 months (14–62 months), only 2 patients on Tinosporin had recurrence. None of the patients needed surgical intervention other than diagnostic biopsy or control of infection, and none received steroids.

**Conclusion:** IGM is a benign, often self-limiting disorder. However, it mimics malignancy in 60% cases, and histology clinches the diagnosis. We report the efficacy of steroid-free management of IGM with immunomodulatory herbal origin phytopharmaceutical drug Tinosporin. It is safe, inexpensive and effective. Large volume excisions or mastectomies can be reserved for severe and refractory cases.

## 1. Introduction

Granulomatous mastitis (GM) is a rare, benign, chronic inflammatory lesion of the breast, frequently seen in premenopausal women, post-childbirth [1]. It was first described in 1972 as a lesion clinically simulating breast carcinoma [2]. It is a distressing condition which often masquerades as breast cancer, leading to diagnostic and therapeutic dilemmas for clinicians, and anxiety for patients. Patients with GM usually present with unilateral, discrete, palpable breast mass or multiple masses, often associated with skin changes, nipple retraction, sinus formation and axillary lymph node enlargement. Histologically referred to as granulomatous lobular mastitis, it is considered as secondary GM when associated with trauma, hyperprolactinaemia, infection (mycobacterial, bacterial or fungal) or sarcoidosis [3, 4]. In the absence of an identifiable cause, it is referred to as primary or idiopathic GM (IGM) [3]. The pathophysiology of IGM is poorly understood, and histologically, it is chronic inflammation of ducts and lobules secondary to stasis of duct secretions. On imaging, over 50% of them resemble malignant lesions [1, 4]. The considerable overlap of clinical features and radiological findings between IGM and malignancy makes the definitive diagnosis difficult, often requiring multiple investigations and repeated biopsies to exclude cancer of the breast [5].

Various lines of management have been described in the literature. Recently, the international multidisciplinary consensus guideline article on GM has provided a comprehensive review of the aetiopathogenesis and provided treatment recommendations and management pathways [6]. The difficulty in the treatment of IGM lies in its indolent course, diagnostic delays, slow response, lack of an effective treatment and high relapse rates. The average treatment duration ranges from 3 to 6 months, sometimes lasting up to years. While some believe it to be a self-limiting condition requiring no treatment, the treatment ranges from antibiotics to immunosuppressants such as steroids, methotrexate and even surgical excision with reconstruction. Some have also used vitamin D supplementation and ozone therapy for the treatment of IGM [7, 8]. The abundant available literature theorising the aetiopathogenesis and management of IGM reflects lack of consensus and variability of practises.

Moreover, the data on the clinicopathological presentation and treatment outcomes in Indian population are scarce. There is an overall practice of using steroids for rapid and acute control of symptoms. However, the relief with steroids is only temporary and recrudescence after stopping steroids is common. Recent studies focus on use of chemotherapeutic agents such as methotrexate [3]. Since IGM is thought to be an immune-mediated inflammatory condition of the breast, hence immunomodulatory agents may be explored in its treatment. *Tinospora cordifolia*, also called as Giloy, Guduchi or Amrita, is a well-known Ayurvedic/herbal product used in ancient Indian Ayurvedic medicine to treat jaundice, rheumatism, skin diseases, diabetes, inflammation and allergies. The pharmacological action of the plant is to its chemical constituents such as diterpenoid lactones, glycosides, steroids, sesquiterpenoid, phenolics, aliphatic compounds, essential oils, a mixture of fatty acids and polysaccharides [9]. We routinely use this drug for the treatment of IGM and rarely use corticosteroids. We retrospectively evaluated the efficacy of Guduchi (in the form of a 250-mg tablet) in our cohort of patients with IGM.

## 2. Materials and Methods

**Figure 1:**
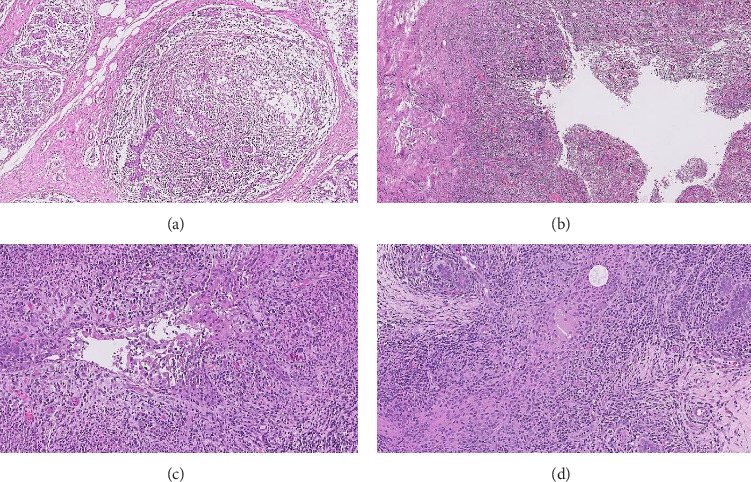
(a) Lobulocentric inflammation, (b) abscess formation, (c) dense mixed acute and chronic peri-ductal inflammation with ductular destruction, and (d) ill-defined granulomas with multinucleate giant cells.

We analysed retrospectively the clinicopathological characteristics of patients diagnosed with IGM on histopathology during January 2018 and December 2022. Data regarding disease presentation, clinical symptoms and signs, radiological findings, treatment patterns and response rates were obtained from electronic medical records of the hospital. All patients had a trucut or incision biopsy. After ruling our malignancy of the breast, the biopsies showing benign breast ducts with lobulocentric mixed inflammation, fat necrosis, histiocytic aggregates forming ill-defined or well-defined noncaseating granulomas, with or without microabscesses were subjected to special stains (including acid fast stain for tuberculosis, Gram stain for bacterial infections and Grocott's methenamine silver (GMS) for fungal infections). After excluding the specific infective aetiologies, in conjunction with histological findings, a diagnosis of IGM was rendered (Figure 1). Whenever appropriate, blood investigation and chest X-ray was done, and physician opinion was sought to rule out extrapulmonary tuberculosis. In case of an acute chronic inflammation, a course of amoxycillin and clavulanic acid was administered first before beginning immunomodulatory treatment. After ruling out active inflammation or tuberculosis and other causes of GM, patients were prescribed empirical broad-spectrum antibiotics such as ciprofloxacin/doxycycline or clindamycin for an initial period of 2 weeks followed by immunomodulatory treatment comprising of Tinosporin capsule for a period of 3–6 months. Patients were followed up at a 3-monthly interval to assess response. Data were collected and analysed in SPSS v-25. Clinicopathological variables were reported as numbers, percentages, and ^∗^. And the chi-square test was applied for comparison of patients' response rates when they received Tinosporin versus when they did not.

## 3. Results

### 3.1. Clinical Presentation

There were a total of 315 patients with histological diagnosis of IGM, of which 132 had complete clinical records and treatment details available. The rest were mainly referred from outside centres for diagnostic purposes, and patients did not receive treatment at our centre and hence were excluded from this analysis. The median age at presentation was 39 years (25–77), and 70% patients were under 45 years of age. Hundred and seven patients (81.7%) were premenopausal. Of these 132 cases, 78 (59.1%) had clinical suspicion of breast cancer. The most common presenting symptom was a breast lump; in 110 patients (83.33%), 23 (17.42%) of them with ulcerations of the overlying skin. Eleven patients (8.3%) presented with discharging sinuses with diffuse lumps, eight (6%) presented with abscesses, while three (2.27%) presented with complaints of breast pain.  Table 1 provides the details of clinicopathological characteristics.

### 3.2. Imaging Findings

On radiological evaluation with mammography and ultrasound (*n* = 132), 84 (63.64%) presented as BIRADS 4 and 5 lesions, raising the suspicion of malignancies. The diagnosis was established by trucut biopsy (blind or USG-guided) in all patients with 21 (15.9%) patients needing an excision/incision biopsy for histological confirmation. On histopathology, 14 of the 132 patients (10.6%) had a diagnosis and had signs of acute inflammation superimposed on IGM in the form of microabscesses, while further 11 (16.9%) were identified to be infective on microbial culture—*Staphylococcus aureus* (4), *Escherichia coli* (3), *Atypical mycobacteria* (2), *Enterococcus* (1) and *Candida* (1) growth, while the others had no organisms.

### 3.3. Treatment Details

**Figure 2:**
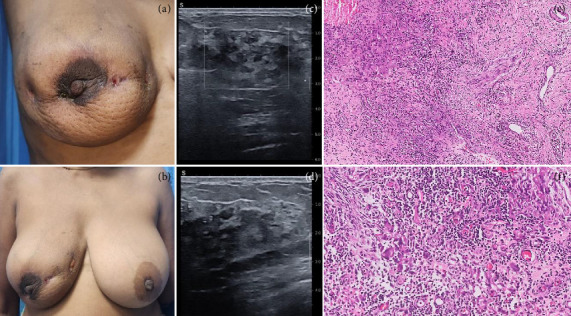
Clinical Scenario 1: 53-year-old perimenopausal lady, diabetic, presented with (a) right breast 12 × 12 cm diffuse lump with overlying peau d'orange and (b) ulcerated skin in the inner central region. Ultrasound showed (c) multiple diffuse masses, hard on elastography, and (d) suspicious lymph nodes, BIRADS 4C. Biopsy showed granulomatous mastitis with histiocytes, negative special stains (ZN, PAS, and GMS). (e) Sheets of multinucleate giant cells with abundant mixed inflammation, granulation tissue, and surrounding fibrosis (H&E, x100). (f) The inflammatory infiltrate comprises of neutrophils, lymphocytes, and plasma cells (H&E, x200).

**Figure 3:**
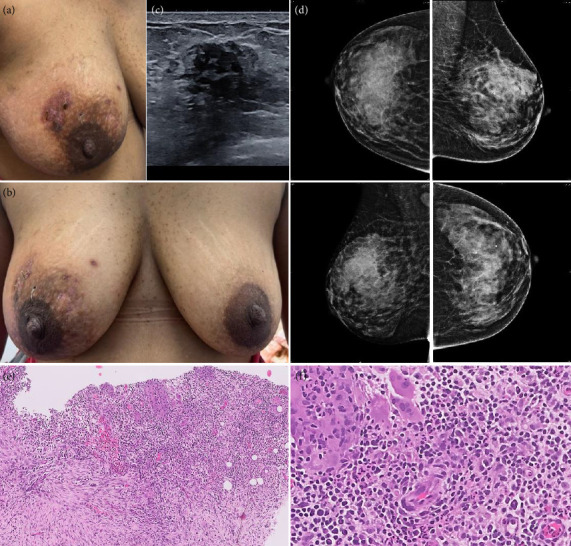
Clinical Scenario 2: 31 year old, premenopausal, presented with (a) right breast 4 × 4 cm diffuse lump with fluctuation in the right upper outer quadrant and (b) skin changes extending upto the lower inner quadrant. Ultrasound showed (c) multiple ill-defined confluent hypoechoic areas, peripheral vascularity, and mobile echoes within, and mammography had (d) global asymmetry and high density lesion in the right upper outer quadrant. BIRADS 4A. Biopsy: granulomatous mastitis. (e) Numerous multinucleate giant cells with surrounding abundant inflammation and fibrosis (H&E, x100). (f) The inflammatory infiltrate comprises of admixed neutrophils, histiocytes, giant cells, lymphocytes, and plasma cells (H&E, x400). She was given a course of doxycycline 100 mg BD for 2 weeks, followed by T Guduchi.

**Figure 4:**
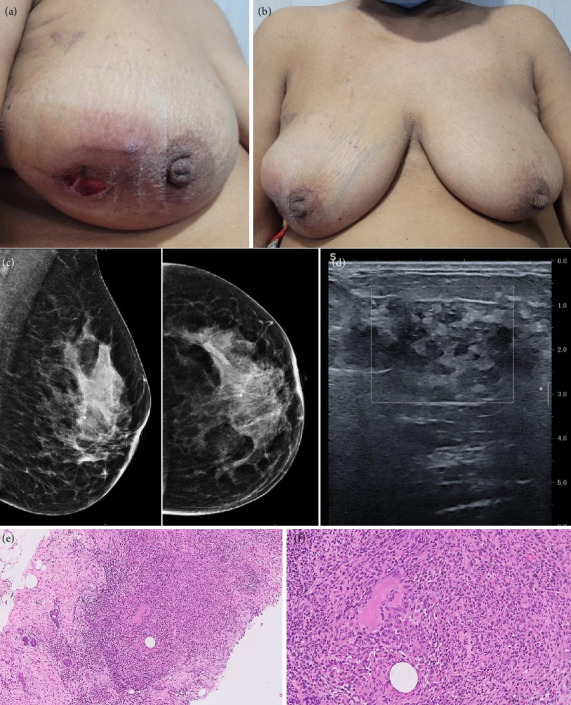
Clinical Scenario 3: 47 year old lady presented with a lump in the right breast since 1 month, (a) skin ulceration and pus discharge with (b) underlying 5 × 5 cm lump in the outer central region of the right breast. On mammography, she had (c) global asymmetry in the right breast with (d) irregular architectural distortion on ultrasound, over an area of 3.9 × 3.8 cm with internal vascularity and surrounding non-mass enhancement on CEM upto 8 cm, BIRADS 4B. A biopsy showed (e) benign breast parenchyma with dense mixed inflammation with fat necrosis and fibrosis (H&E, x100). (f) The mixed inflammation comprises of multinucleate giant cells, neutrophilic microabscesses and lymphocytes (H&E, x200).

Antibiotics were prescribed to 101 patients. 13 patients did not follow up, and hence their compliance with respect to medication was not documented. Steroids were only prescribed to 1 patient for rapid control of symptoms. None of the patients were prescribed any other immunosuppressant or chemotherapeutic drugs. Tinosporin tablet (500 mg Guduchi stem extract per day) was prescribed to 91 patients. For 13 patients, the data on Tinosporin prescription were missing as the patients did not follow up. The treatment duration ranged from 1 to 7 months, with 75 (97.4%) patients requiring minimum 3 months of treatment. Symptomatic relief was seen in 72/77 patients (93.5%) who were prescribed Tinosporin. Only 5 patients did not achieve response, while data were missing for 13 patients. Two patients on Tinosporin had recurrence of symptoms. Only five patients, those who did not respond to Tinosporin (6.5%), were treated by surgical curettage and excision. Of the 41 patients who were not prescribed Tinosporin, 13 patients had defaulted on the prescribed treatment. Of the 28 with follow-up, 8 were given anti-tuberculous treatment due to the suspicion of tuberculosis prior to visiting our centre, while the others were treated with antibiotics and/or observation, and one was given steroids. Only 11 of the 28 patients (39.29%) responded to this form of treatment. Treatment details are summarised in Table 2, and response to treatment in Table 3. We summarise the clinical presentations, radiological and histopathological findings of three patients in the series in Figures 2, 3, and 4.

## 4. Discussion

We present a large dataset of 132 patients presenting with IGM treated with a steroid-free regimen without surgical resection. The main contention behind diagnosis and work up of these patients had been to rule out malignancy since more than 60% had a clinicoradiological suspicion of breast cancer, as IGM also commonly presents as a painless breast mass. Azizi et al. reported a series of 474 patients in whom 70% patients presented with a palpable lump [10]. Another series of 206 patients from Iran had 81% patients presenting with a suspicious breast mass [11]. There can be subsequent rapid progression of inflammation, usually from periphery to areola due to the involvement of lobules in its pathogenesis. Subcutaneous abscesses form which lead to fistulae and sinuses in severe cases. In our cohort, 110 patients (83.33%) presented with a suspicious lump, amongst which 38 (28.8%) had features of locally advanced breast cancers. Rarely, associated systemic manifestations such as fever and erythema nodosum or polyarthritis are seen [10, 12–15].

The diagnostic dilemma of IGM extends to radiology as well. Sixty-three percent of our patients had radiologically suspicious findings suggestive of malignancy, of which 5.3% had BIRADS 5 lesions. Mammography is often difficult due to acute inflammation, abscesses and discharging sinuses, commonly has vague findings such as focal or global asymmetric density, blurred edges, skin thickening and parenchymal distortion [16]. 81.1% of our patients were premenopausal, consistent with the literature [1], mammography may not be helpful in this population as they have dense breast parenchyma. The most common finding was a hyperdense mass (35, 43.75%), while over half the patients had only subtle architectural distortion (15, 18.75%) and asymmetric densities (15, 18.75%). Four (5%) patients had only benign appearing calcifications. The literature suggests MRI may often not add value to the diagnosis [17]. Ultrasound is the first line investigation as it can detect abscesses and sinuses, guide biopsies and is effective for follow-up response. The most common finding was an irregular hypoechoic mass (44.1%). Eighteen patients (15.3%) had only an area of architectural distortion, and 18 (15.3%) only ill-defined hypoechoic areas with surrounding hyperechogenic regions. Hence, over 50% patients had vague findings even on imaging, while 13% had completely normal mammography and ultrasound, emphasising that IGM may be completely occult, needing a high index of suspicion and requiring a multidisciplinary discussion for conclusive diagnosis.

The aetiology of IGM remains obscure. It may be primary or autoimmune (idiopathic) or may be secondary to infection which is difficult to document. The pathophysiology involves a hypertrophic state after pregnancy and lactation. The ductal permeability increases, and retained milk due to stasis which is immunogenic enters the lobular mesenchyme, causing a cellular immune response and granuloma formation [18]. Poor breastfeeding and weaning habits are hence a risk factor [12, 19]. Autoimmunity, infections, smoking and *α* 1-antitrypsin deficiency play a role in the pathogenesis [20]. Obesity, use of hormonal therapy, pregnancy and lactation have been aetiologically related in the affected patients [4]. Microbial infection with corynebacterium and *Staphylococcus* is also causatively associated with IGM, both often found in the cultures of surgical specimens and biopsies [21–24]. Atypical mycobacteria, seen less commonly, must be differentiated from tuberculous mastitis. Pathologically, IGM manifests as lobulocentric granulomatous inflammation with histiocytic aggregates forming ill-defined or well-defined noncaseating granulomas. Multinucleate giant cells, lymphocytes, plasma cells, eosinophils and fat necrosis may be seen, replete with neutrophils, which form microabscesses in case of superimposed acute inflammation. Lobulocentricity may be masked in cases with extensive inflammation (Figure 1). This often requires a generous tissue sample and warrants a confirmatory incision or excision biopsy sample to accurately rule out malignancy and tuberculosis. A diagnostic algorithm based on a multidisciplinary panel of 66 experts from 509 articles, suggested ruling out hyperprolactinaemia, followed by simultaneous ultrasound-guided tissue biopsy and cultures of aspirates, for all patients with clinical suspicion of IGM. They recommended the addition of antibiotics for patients with positive cultures, in addition to the primary management, which included observation, steroids or surgery based on the severity of symptoms [6]. At our institution, biopsies showing features suggestive of IGM were subjected to special stains (including acid fast stain for tuberculosis, Gram stain for bacterial infections and GMS for fungal infections). After excluding the aforementioned specific infective aetiologies, in conjunction with histological findings, a diagnosis of IGM was rendered. In 91% patients tested, we found no growth on culture and hence no causative pathogen was identifiable to guide specific antibiotic treatment.

IGM is a rare condition, with poor efficacy and durability of available treatment and a prolonged and recurrent course. It is often self-limiting, but remission is slow and recrudescence is commonly seen during therapy. Only 50% patients achieve remission at 2–24 months, taking on average 5 months for relief [25, 26]. If an identifiable cause is found such as infection or hyperprolactinaemia, treatment must be directed towards it. Bromocriptine and cessation of drugs causing hyperprolactinaemia (e.g., antipsychotics) are effective. IGM is commonly treated with antibiotics based on culture sensitivity tests. An empirical treatment with clindamycin, levofloxacin or azithromycin can be used before sensitivity is available or if the culture report does not identify an organism [27, 28]. Some have found rifampicin 300 mg twice daily for 6–9 months effective with low recurrence; however, the side effects of rifampicin can cause morbidity [13]. In our series, 101 patients were prescribed antibiotics, namely, ciprofloxacin, clindamycin or doxycycline, and only 11 (39.29%) responded to antibiotics and observation alone. Overall, the efficacy of antibiotics alone in the management of IGM is poor, ranging from 3% to 50% response and with 23% relapse rate [4, 11].

The most commonly reported first-line treatment is oral corticosteroids, with a response rate of 64%–80% [11, 29], but requiring a mean treatment duration of 4–6 months (range, 1–12 months) [30] and a high relapse rate of 30%–40% [30, 31]. There is no consensus on the ideal duration of steroids, commonly leading to months of use up until complete clinical response due to the slow response rate [19]. Prolonged use of corticosteroids is associated with weight gain, osteoporosis and infections among other side effects. High-dose steroid regimens, intralesional and topical steroids have been explored to circumvent the systemic toxicities, some have found a lower recurrence in the local treatment group compared with oral corticosteroids (8 versus 46%) [32, 33]. One study compared steroid use with conservative management and found that steroid use did not significantly reduce the mean recovery period [1]. For those resistant to steroids, having contraindications, or suffering from their side effects, other immunosuppressive agents have been described. Methotrexate is commonly used, sometimes in combination with corticosteroids to reduce the dose [3, 34, 35]. The treatment duration remains 6–24 months with a dosage of 5–15 mg per week [36]. Methotrexate also has side effects and must be advised with precaution in these women of child-bearing age. Yuan et al. described repeated aspirations and saline washes with injection of 40 mg triamcinolone acetate 6-7 times and showed 78% efficacy [6, 37]. Surgical wide excision may be required in those with complicated local sinuses, fistulae and abscesses for those not responding to treatment. A recent meta-analysis found surgery to have a high cure rate and low recurrence rate at par with that of steroid use [19]. However, a prospective study from Kolkata found high rate of relapse with surgical excision alone [38]. Surgery is contraindicated in acute inflammation and extensive involvement of the breast skin. Recurrences are common in IGM, 15.4%–24.8%, and are diagnosed clinically [39–42]. The clinical presentation remains the same and often worsens in relapses. Young age, corynebacterium on culture and pregnancy are at higher risk of recurrence and need longer treatment durations [39].

None of the above commonly practiced treatments result in an effective and durable remission. Immunological dysfunction plays a major role in the pathogenesis of IGM. Alterations in intracellular cytokine expression patterns in T-lymphocyte subsets after ozone therapy in refractory IGM have been found [8]. Deng et al. found predominant presence of CD3, CD4, CD8, CD79a, IgG and IgM, highlighting the role of cell-mediated immunity [43]. A Turkish study evaluated molecular changes at the proteome level, extracts from IGM patients had a significant overlap with immune related proteins, and those involved in cancer metabolism [44]. This called for exploration of new avenues in the treatment, with a scientific approach based on the immunological pathogenesis, while avoiding drugs that cause significant side effects with a variable and often negligible impact to the course of the disease. The response to steroids and immunosuppressants supports this theory [43].

*Tinospora cordifolia*, commonly named “Guduchi” in Sanskrit, belonging to the family Menispermaceae is a genetically diverse, large, deciduous climbing shrub found at a higher altitude, with green-yellow flowers [45–47]. The active components include alkaloids, steroids, diterpenoid lactones, aliphatics and glycosides, 11-hydroxymustakone, cordifolioside A, magnoflorine, N-methyl-2-pyrrolidone, N-formylannonain tinocordiside and syringin [48–50], from different parts of the plant body, including the root and stem. It has recently been reported antidiabetic, antispasmodic, anti-inflammatory, antiarthritic, antioxidant, antiallergic, antileprotic, antimalarial, hepatoprotective and antineoplastic too. Tinosporin, extracted from the stem root, is the active ingredient effective against IGM. There are multiple documented mechanisms of action of Tinosporin. As per an analysis of 175 articles, active compounds [49, 50] have potential immunomodulatory and cytotoxic effects [51–54]. They boost the phagocytic activity of macrophages, production of reactive oxygen species in neutrophils [55], and enhance nitric oxide [56]. In mice, extracts cause upregulation of IL-6 cytokine, activation of cytotoxic T-cells and B-cell differentiation [57]. The (1,4)-alpha-d-glucan in *Tinospora cordifolia* activates human lymphocytes with downstream synthesis of pro- and anti-inflammatory cytokines in vitro [58]. It scavenges free radicals, lowers thiobarbituric acid reactive substances levels and increases antioxidants glutathione, superoxide dismutase, ascorbic acid and glutathione S-transferase in kidney, preventing nephropathy [59]. Oral administration of plant extracts prevented the occurrence of liver damage in mice models. Decreased SOD was observed in mice suffering from lead toxicity [60]. The methanol extracts have potential antimicrobial action [59] against *E. coli*, *S. aureus* and other Gram-positive bacteria. Intramammary infusion of hydromethanolic extracts of Tinospora showed enhanced phagocytic activity of polymorphonuclear cells in bovine subclinical mastitis [61, 62].

We have been treating IGM primarily with immunomodulators. Usually following a course of empirical or culture-based antibiotics for 7–14 days in those with signs of inflammation, our patients are prescribed oral Tinosporin tablet 500 mg for three months. At 3 months, the patients are asked to visit for a clinical follow-up, wherein the response is assessed based on symptoms, clinical features and size of the lump. In case of good response, treatment with Guduchi is continued for another 3 months. In case of persistence of symptoms or progression, the tablets are continued longer as per clinicians' discretion. Steroids are seldom used.

In our audit, 97.4% patients responded to a maximum of 3 months of treatment with Tinosporin, with a 93.5% (72/77 patients) response rate. Only five patients (6.5%) did not respond to the treatment and were treated with cavity curettage, and two patients had a relapse, with a median follow-up of 36 months. Only 11 of the 28 patients (39.29%) who were treated without Tinosporin but with antibiotics and observation alone, experienced relief of symptoms. None of the patients suffered from any side effects of the medication or required discontinuation or dose adjustment, or required steroids. None of our patients were prescribed methotrexate or any other immunosuppressants.

The strength of our study is that we provide a safe, steroid-sparing, inexpensive, well-tolerated and effective treatment regimen for IGM without the side effects which are often seen with steroids and without surgical resections amounting to partial or total mastectomy. The limitations of the study are its retrospective nature and lack of comparison with different treatment strategies. The loss to follow-up of patients inherent to a retrospective design weakens the data. Future studies are needed to further understand the aetiopathogenesis of this disease entity and determine the factors causing it.

## 5. Conclusion

IGM is a benign inflammatory disorder of the breast. We provide an effective treatment strategy for IGM with a natural plant product Tinosporin without steroids and surgical resections.

## Figures and Tables

**Table 1:** Clinicopathological profile of 132 cases of IGM.

Demographic category	*N* = 132	%
Age (median 39 [25–77])		
< 45	93	70.5
> 45	39	29.5
Menstrual status		
Premenopausal	107	81.1
Postmenopausal	25	18.9
Clinical presentation		
Benign	54	40.9
Malignant	78	59.1
Radiological presentation		
Benign	48	36.4
Suspicious	84	63.6
Biopsy		
Core biopsy	36	27.27
Incision biopsy	69	52.27
Diagnostic excision biopsy required	15	11.36
Cultures (*n* = 61)		
Growth	11	18.1
No growth	50	81.9

**Table 2:** Treatment pattern.

Category	*N* = 132	%
Antibiotics		
Prescribed	101	76.5
Not prescribed	18	13.6
Defaulted	13	9.9
Tinosporin		
Prescribed	91	68.9
Not prescribed	28	21.2
Defaulted	13	9.9
Steroids	1	0.8
Surgical excision	5	3.8

**Table 3:** Response to tinosporin.

	Response	No response	Lost to follow-up
Tinosporin (91)	72	5	14
No tinosporin (41)	11	17	13

## Data Availability

The data that support the findings of this study are available from the corresponding author upon request.
